# Magnetic Properties in Mn-Doped *δ*-MoN: A Systematic Density Functional Theory Study

**DOI:** 10.3390/nano12050747

**Published:** 2022-02-23

**Authors:** Keda Wang, Jing Yu, Caixia Chi, Guiling Zhang

**Affiliations:** 1 College of Food and Pharmaceutical Engineering, Suihua University, Suihua 152061, China; shxywangkd@163.com (K.W.); chicaixia617@163.com (C.C.); 2 Key Laboratory of Environmental Catalysis and Energy Storage Materials of Heilongjiang Province, Suihua 152061, China; 3 College of Materials Science and Chemical Engineering, Harbin University of Science and Technology, Harbin 150080, China

**Keywords:** Mn-doped, *δ*-MoN, magnetic properties, theoretical study

## Abstract

Due to the potential applications of transition metal nitrides in modern electronic and spintronic devices, we have systematically studied the magnetic properties of *δ*-MoN induced by the Mn dopant, with the goal of identifying the origin of magnetism and figuring out the magnetic coupling mechanism between the Mn dopants. Based on the density functional theory, one Mn atom doped at different Mo sites (2*a* and 6*c* in the International Tables) in the unit cell of *δ*-MoN was firstly studied. It was found that the Mn dopant located at the 2*a* or 6*c* site leads to significant spin splitting of the density of states, suggesting that the Mn doping induces magnetism in *δ*-MoN. The calculations were then extended to a 2 × 1 × 2 supercell, which contains two impurity Mn atoms. Detailed analysis reveals that the different couplings of the Mn–Mn pair cannot be simply attributed to the different Mn–Mn distances but are closely related to the electronic processes that take place in the segment (–N– or –N–Mo–N–) that connects two Mn dopants. The mechanisms responsible for the FM/AFM coupling of the Mn–Mn pairs are the superexchange and the *p*–*d* exchange mediated by the N atoms, and the *d*–*d* coupling between the host Mo atom and the Mn dopant.

## 1. Introduction

Transition metal nitrides, by virtue of their unique electronic structure and exact nature of bonding (mixed metallic, ionic, and covalent bonding), possess excellent physical properties, such as high hardness [1,2], high melting point [3], and high electronic conductivity [4,5], which have made them attract tremendous attentions in recent years. In order to enhance the performance of this class of materials, much effort has been devoted to investigating the possibility of improving the energetic [6,7,8], mechanical [9,10], and electrical [11] properties by doping foreign atoms. Recent research has confirmed that introduction of foreign transition metal atoms (for instance, Fe, V, Mn, Cr, etc.) into the host material could effectively enhance the magnetism of transition metal nitrides, such as ScN [12,13], CrN [14,15], and ZnN [16,17]. It has been found that the V, Cr, Mn, Fe, Co, and Ni doping can induce magnetism in ScN [12], and the Mn-doped ScN is a dilute magnetic semiconductor with the Curie temperature above 400 K [18,19]. The CrN undergoes a relative increase in the magnetic order with the substitution of Mn atom [14], and V dopant in CrN could introduce holes into the host material and produce a series of inhomogeneous magnetic or electronic states [15]. The ZnN doped with Cr impurity is found to exhibit half-metallic ferromagnetism, which is a good candidate for spintronic applications [16]. It is known that the enhancement or the inducement of magnetism caused by the transition metal doping is closely related to the modification of electronic states; so, if we want to clearly understand the origin of magnetism, it is necessary to investigate the modified electronic structure of the doped transition metal nitrides.

As a typical type of transition metal nitrides, molybdenum nitrides are known to have a set of interesting properties, such as low compressibility [20], high melting point [21], and excellent catalytic activity [22,23], which are very attractive for a wide range of technological applications. Furthermore, their multiple transport properties (including the metallic and magnetic properties [24,25] and, especially, the high *T*_C_ superconductivity [26,27]), also support the potential applications in electronic devices. Molybdenum nitrides can crystallize in different phases, including a stoichiometric structure, hexagonal *δ*-MoN, and two nonstoichiometric structures, cubic *γ*-Mo_2_N and tetragonal *β*-Mo_2_N [27,28,29,30]. In particular, hexagonal *δ*-MoN is considered the hardest superconducting metal nitride [1], whose bulk modulus is measured to be 345 GPa [20] and *T*_C_ is up to 12–15 K [31,32]. A number of experimental works have been performed which mainly focus on the synthesis of pure *δ*-MoN with desired superconducting properties [27,31,33]. Only a few theoretical studies have been devoted to *δ*-MoN with the purpose of investigating its structural, electronic, and mechanical properties [34,35]. Up to now, research concerning the magnetism of *δ*-MoN has been really scarce. Our previous work has theoretically studied the Cr-doping effect on the magnetic and spin transport properties of *δ*-MoN [36]. However, to the best of our knowledge, no theoretical works have been related to the magnetism of *δ*-MoN induced by the Mn dopant.

In this paper, we systematically studied the electronic and magnetic properties of the Mn-doped *δ*-MoN, with the goal of identifying the origin of magnetism and figuring out the magnetic coupling mechanism between the Mn dopants. This paper is organized as follows: Firstly, one Mn atom, with *d*^5^ structure, was used as dopant to substitute one Mo atom located at two different nonequivalent positions (2*a* and 6*c* in the International Tables) in the unit cell, respectively, to investigate the Mn-doping effects on the structural, electronic, and magnetic properties of *δ*-MoN. Then, the calculations were extended to a large supercell with two substitutional Mn atoms to explore the favorable coupling between the Mn dopants. Based on the detailed analysis of magnetic ordering, electronic structure, and spin charge density distribution, the origin of magnetism and the coupling mechanism between the Mn dopants were revealed.

## 2. Computational Details

The calculations were performed using the projector augmented wave (PAW) pseudopotentials as implemented in Vienna ab initio simulation package (VASP) [37]. The exchange correlation functional was treated by Perdew–Burke–Ernzerhof form generalized gradient approximation (GGA-PBE) [38]. Considering the electron correlations in the transition metal *d* shell, we introduce the GGA + *U* method by a simplified approach of Dudarev et al. [39], where the effective Hubbard parameter *U*_eff_ = *U* − *J*. The Hubbard parameter *U* measures the increase in energy caused by placing an additional electron into a particular site, and *J* is a screened Stoner-like exchange parameter. Here, we choose *U*_eff_ = 4 eV for Mn-3*d* electrons [40,41] and *U*_eff_ = 2 eV for Mo-4*d* electrons [42], which are taken from the literature. The tetrahedron method with Blöchl corrections was used to determine the partial occupancies for setting each wave function [43]. The Kohn–Sham orbitals were expanded in a plane-wave basis with a cutoff energy of 500 eV. The Brillouin-zone integration was performed on well-converged 7 × 7 × 7 and 3 × 5 × 3 Monkhorst–Pack *k*-point meshes for 1 × 1 × 1 unit cell and 2 × 1 × 2 supercell, respectively. Both the atomic positions and the unit cell parameters were fully relaxed until the forces on each atom were less than 0.01 eV·Å^−1^. All the calculations were performed based on the optimized geometries.

## 3. Results and Discussions

**Figure 1:**
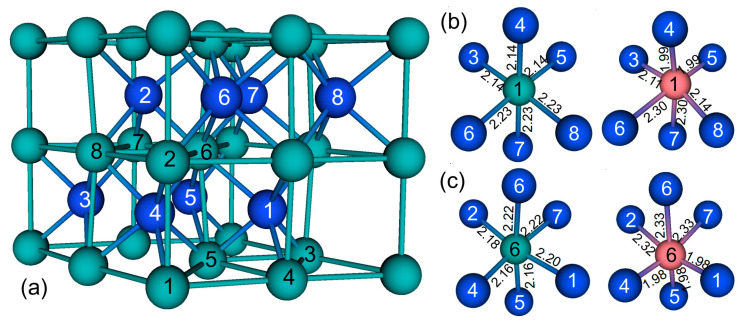
(**a**) Schematic structure of the unit cell of *δ*-MoN, and optimized bond lengths of (**b**) Mn_1_–N and (**c**) Mn_6_–N in Mn-MoN(2*a*) and Mn-MoN(6*c*), respectively. For comparison, the relaxed bond lengths of Mo_1_–N and Mo_6_–N in the pure *δ*-MoN are also presented in (**b**,**c**), respectively. The pink, green, and blue balls denote the Mn, Mo, and N atoms, respectively.

We begin our discussion with the structural, electronic, and magnetic properties of *δ*-MoN, with a single substitutional Mn atom. Hexagonal *δ*-MoN crystallizes in a distorted NiAs-type structure with a space group of *P*6_3_*mc* (186), which has been determined by experiment investigations [20,32,44], as well as theoretical calculations [34]. In *δ*-MoN, Mo atoms have two kinds of lattice points: one is labeled 2*a* and the other is labeled 6*c* in the International Tables. Each unit cell contains eight Mo atoms and eight N atoms. The unit cell of *δ*-MoN is presented in Figure 1a, in which the Mo atoms (green balls) are numbered to facilitate our discussion. Mo_1_ and Mo_2_ are in the 2*a* (0, 0, 0) sites, while Mo_3_–Mo_8_ are in the 6*c* (0.5082, 0.0165, −0.0064) sites [32]. To find the preferable location of Mn impurity in *δ*-MoN, we substitute one Mo atom with one Mn atom at two different nonequivalent Mo sites (2*a* and 6*c*), respectively. Here, the side Mo_1_ atom (representing for the 2*a* site) and the central Mo_6_ atom (representing for the 6*c* site) were substituted, respectively, by one Mn atom to carry out VASP calculations for investigating the Mn-doping effects on the electronic and magnetic properties of *δ*-MoN. Such a Mn-doped unit cell contains one Mn atom, seven Mo atoms, and eight N atoms, corresponding to the Mn doping concentration of 12.5%. Following that, the two doped systems with Mn doping at 2*a* and 6*c* sites are written as Mn-MoN(2*a*) and Mn-MoN(6*c*), respectively.

Firstly, we performed a geometry optimization for the pure *δ*-MoN. It is found that the calculated lattice constants of *δ*-MoN (*a* = 5.757 Å, *c* = 5.668 Å) are in good agreement with the experimental values [32] with deviations within 0.9%. On the basis of the equilibrium structure of *δ*-MoN, the cells of Mn-MoN(2*a*) and Mn-MoN(6*c*) were constructed and relaxed to the minimum energy configurations. The optimized lattice parameters and bond lengths are presented in Table 1 and Figure 1b,c, respectively. The presence of Mn has a marked effect upon the geometry of *δ*-MoN. After relaxation, the substitutional Mn_1_/Mn_6_ atom is found to pull some N atoms closer and push some N atoms farther, suggesting that the lattice structure at the Mn site is distorted and forms a new local structure. The bond lengths of Mn_1_–N and Mn_6_–N are in the range of 1.99–2.30 Å and 1.98–2.33 Å for Mn-MoN(2*a*) and Mn-MoN(6*c*), respectively, which are different from those of Mo_1_–N (2.14–2.23 Å) and Mo_6_–N (2.16–2.22 Å) in the pure *δ*-MoN. The shortest bond lengths occur at Mn_1_–N_4,5_ (1.99 Å) for Mn-MoN(2*a*) and Mn_6_–N_1,4,5_ (1.98 Å) for Mn-MoN(6*c*), which are very close to the sum of the covalent radius of Mn^3+^ and N^3−^ (1.92 Å), indicating a covalent bonding feature between Mn and its nearest N atoms. The average bond lengths of Mn_1_–N and Mn_6_–N are 2.14 Å and 2.15 Å, respectively, which are about 0.05 Å and 0.04 Å shorter than those of Mo_1_–N and Mo_6_–N. The shortening of the average Mn_1_–N/Mn_6_–N bond length consequently leads to the shrinkage of the local structure, and thus results in the decrease of the lattice parameters of Mn-MoN(2*a*)/Mn-MoN(6*c*), compared to that of *δ*-MoN (cf. Table 1). This case is probably due to the fact that the radius of Mn^3+^ is smaller than that of Mo^3+^.

**Figure 2:**
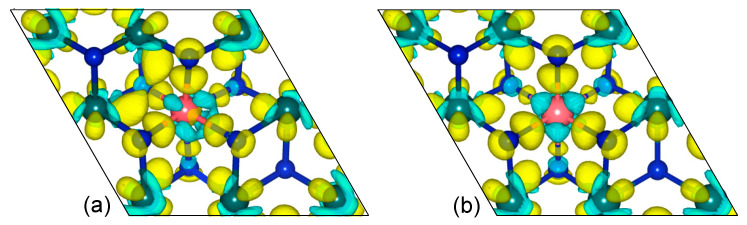
Charge density difference isosurfaces for (**a**) Mn-MoN(2*a*) and (**b**) Mn-MoN(6*c*). The pink, green, and blue balls denote the Mn, Mo, and N atoms, respectively.

The charge transfer between Mn dopant and *δ*-MoN can be examined by the charge density difference, which can be determined by subtracting the charge densities of pristine *δ*-MoN and isolated Mn atom from the total charge density of the Mn-doped system. Figure 2 shows the charge density difference isosurfaces of Mn-MoN(2*a*) and Mn-MoN(6*c*), which describe redistribution of the valence charge density of atoms caused by chemistry bonding. The yellow and light blue regions refer to electron accumulation and depletion, respectively. Evident charge depletion around the Mn atom can be observed from the isosurfaces. Integrating the density of states up to the Fermi energy, the estimated charges transferred from Mn to *δ*-MoN are 1.22 and 1.12 electrons for Mn-MoN(2*a*) and Mn-MoN(6*c*), respectively. The charge accumulation between Mn and N suggests a strong covalent character of the Mn–N bonds, which is in line with the calculated bond lengths, as discussed above. This may be generated by the *p*–*d* hybridization between Mn and N, which can be further confirmed by the partial density of states.

The preferable site for the Mn dopant in *δ*-MoN can be determined by estimating the formation energies of different doping configurations. The formation energies of Mn-MoN(2*a*) and Mn-MoN(6*c*), $E_{f}$, are calculated by the following formula [45]:

(1) $$E_{f}=E_{\mathrm{tot}}\left(\mathrm{doped}\right)\;-\;E_{\mathrm{tot}}\left(\mathrm{pure}\right)\;+\;m\mu_{\mathrm{Mo}}-n\mu_{\mathrm{Mn}}$$

where *E*_tot_(doped) and *E*_tot_(pure) are the total energies of the Mn-doped and undoped *δ*-MoN, respectively. $\mu_{\mathrm{Mo}}$($\mu_{\mathrm{Mn}}$) is the chemical potential of the Mo (Mn) atom. The *n*, *m* are the numbers of the doped Mn atoms and the substituted Mo atoms, respectively. The calculated formation energies of the Mn-doped systems are listed in Table 1. Note that the formation energies of Mn-MoN(2*a*) and Mn-MoN(6*c*) decrease relative to the pure *δ*-MoN. A negative energy means that the formation of Mn-MoN(2*a*)/Mn-MoN(6*c*) is spontaneous, that is, the Mn dopant will easily occupy the 2*a*/6*c* Mo lattice site in *δ*-MoN. The relative stability of two Mn-doped configurations can be evaluated by comparing their formation energies. The smaller the formation energy, the more stable the structure. As seen from Table 1, the formation energy of Mn-MoN(2*a*) is slightly lower than that of Mn-MoN(6*c*). It seems that the Mn dopant prefers to substitute the 2*a* Mo lattice site in *δ*-MoN, and Mn-MoN(2*a*) is a more energetically favorable structure.

**Figure 3:**
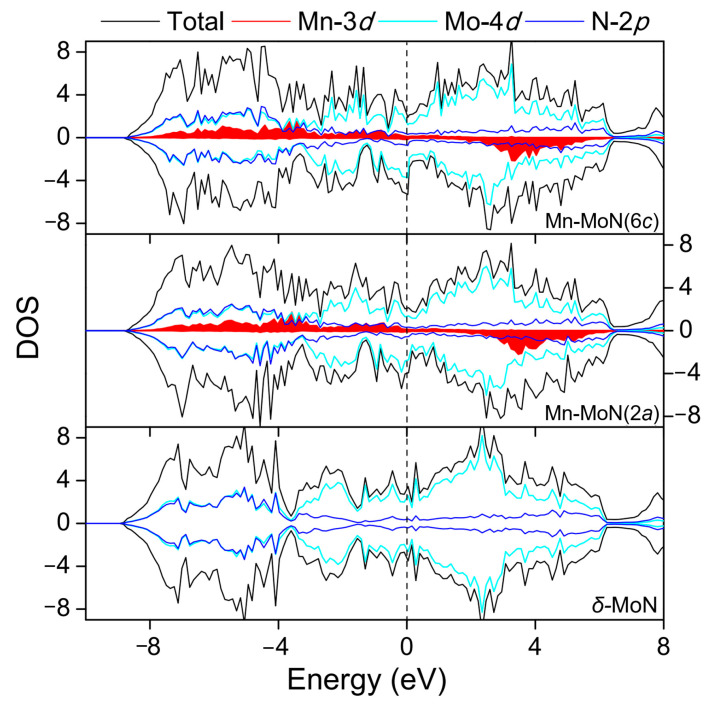
Total and partial DOS of *δ*-MoN, Mn-MoN(2*a*), and Mn-MoN(6*c*). The Fermi level is set as zero.

To investigate the effect of Mn dopant on the modification of electronic structure, the spin-polarized density of states (DOS) were calculated for both of the undoped and doped systems, as given in Figure 3. The characteristic of the density of states around the Femi level demonstrates the metallic nature of these systems. It is seen that the majority and minority spin carriers of the pure *δ*-MoN exhibit mirror symmetry, demonstrating its nonmagnetic characteristic. However, evident spin-polarization of the density of states is observed in the Mn-doped systems. This asymmetrical distribution of the wave functions of the spin-up and spin-down channels on the total DOS of Mn-MoN(2*a*) and Mn-MoN(6*c*) suggests that the Mn dopant induces magnetism in *δ*-MoN. The Mn substitutions at 2*a* and 6*c* sites result in 2.74 and 2.56 *μ*_B_ total magnetic moments for Mn-MoN(2*a*) and Mn-MoN(6*c*), respectively. The partial DOS analysis reveals that the magnetism of the Mn-doped systems is mainly due to the presence of Mn impurity in the *δ*-MoN host, as well as a partial contribution from the neighboring Mo and N atoms around the dopant. A local magnetic moment of about 4 *μ*_B_ (4.03 *μ*_B_ for Mn_1_ and 3.99 *μ*_B_ for Mn_6_) per Mn atom obtained from the calculations indicates that the Mn ion presents a Mn^3+^ valence state (3*d*^4^). It is notable from the partial DOS that significant hybridization occurs between the orbitals of Mn-3*d*, Mo-4*d,* and N-2*p*, which is a good proof of the much more delocalized Mn impurity bands in comparison to an isolated Mn atom. The Mn-3*d* states spread throughout the valence band and the conduction band, with almost completely filled majority *d* bands and nearly unoccupied minority *d* bands. The strong interactions between Mn and Mo induce evident spin polarization of the Mo atoms with large magnetization. The Mo atoms adjacent to the Mn dopant get as large as −0.51 and −0.47 *μ*_B_-induced magnetic moments for Mn-MoN(2*a*) and Mn-MoN(6*c*), respectively. The neighboring N atoms to the Mn dopant are also slightly spin-polarized with small magnetic moments of −0.03 to 0.02 *µ*_B_.

**Figure 4:**
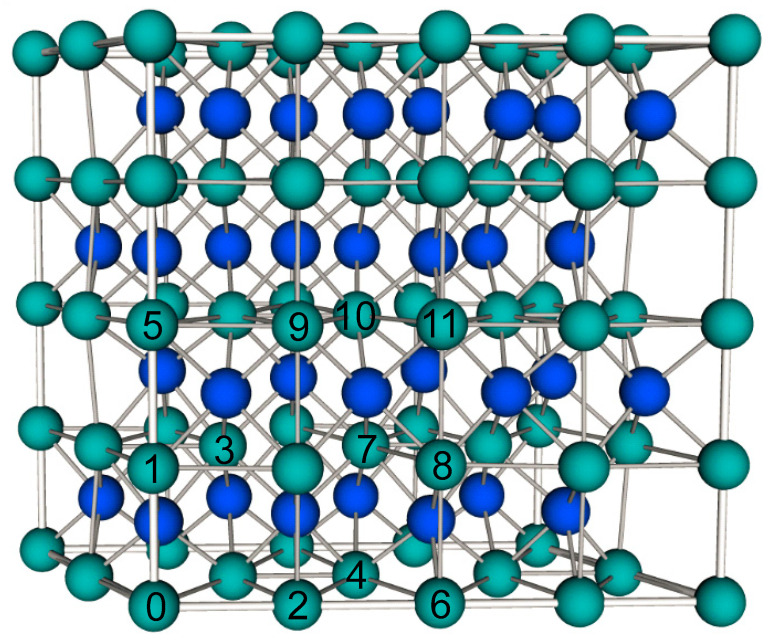
A 2 × 1 × 2 supercell structure of *δ*-MoN. With respect to one Mn atom (labeled as 0), we select eleven nonequivalent positions for the other Mn atom (as numbered from 1 to 11) based on the symmetry of the supercell. The green and blue balls denote the Mo and N atoms, respectively.

It is necessary to investigate the favorable coupling between the Mn dopants. Thus, we extend the analysis to a pair of Mn atoms in *δ*-MoN. Here, a 2 × 1 × 2 *δ*-MoN supercell, as shown in Figure 4, was used to investigate the magnetic coupling between two impurity Mn atoms. This supercell contains 32 formula units of MoN, in which two of the Mo atoms are replaced by two magnetic Mn atoms, to form a supercell containing 2 Mn atoms, 30 Mo atoms, and 32 N atoms, amounting to 6.25% Mn doping concentration. Such a large supercell adopted in the calculations allows us to simulate various distributions of two Mn dopants. In brief, we fixed one dopant Mn atom and varied the possible positions of the second Mn atom. As shown in Figure 4, the first Mn atom is fixed at a Mo lattice site (marked 0), and the second Mn atom changes its doping position from 1 to 11 to form the Mn–Mn pair in the supercell. The substituted Mo atoms numbered in 1–11 follow the sequence of the distance to Mo_0_. This generates eleven different doping configurations, in which the Mn–Mn separation within the supercell varies from 2.73 to 8.08 Å. Hereafter, these arrangements are referred to as the (0, *n*) configurations.

After geometry optimization, we calculated the formation energies for all the eleven nonequivalent configurations based on Equation (1), and the results are presented in the eighth column of Table 2. The formation energy for the single-doped system, in which is one single Mn atom doped at 0 Mo lattice site, is also calculated for comparison. It is observed from Table 2 that the formation energies of these double-doped systems vary from −3.06 to −3.48 eV, which is much lower than that of the single-doped system (−1.73 eV). This indicates that the formation energy of Mn decreases with its doping concentration, and it is highly favorable to form Mn substitution in *δ*-MoN.

**Figure 5:**
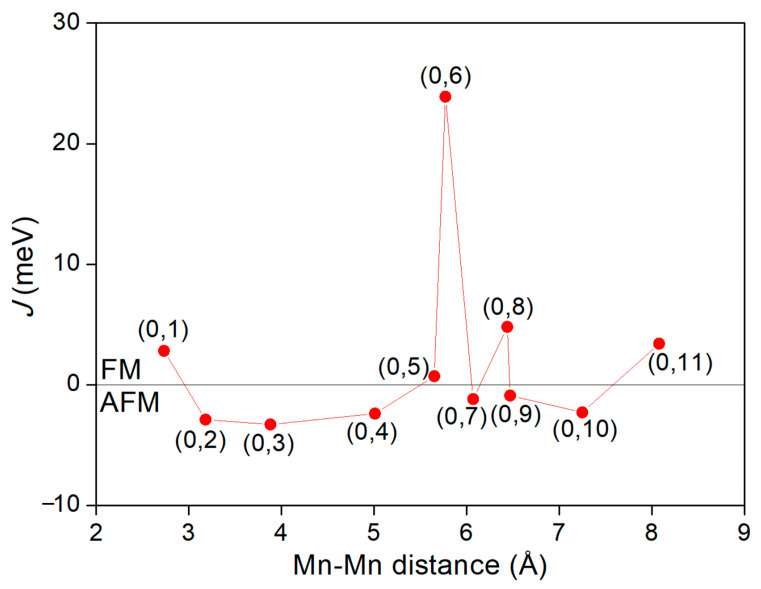
Variation of *J* value as a function of Mn–Mn distance. The positive value of *J* represents the FM state, while the negative value represents the AFM state.

**Figure 6:**
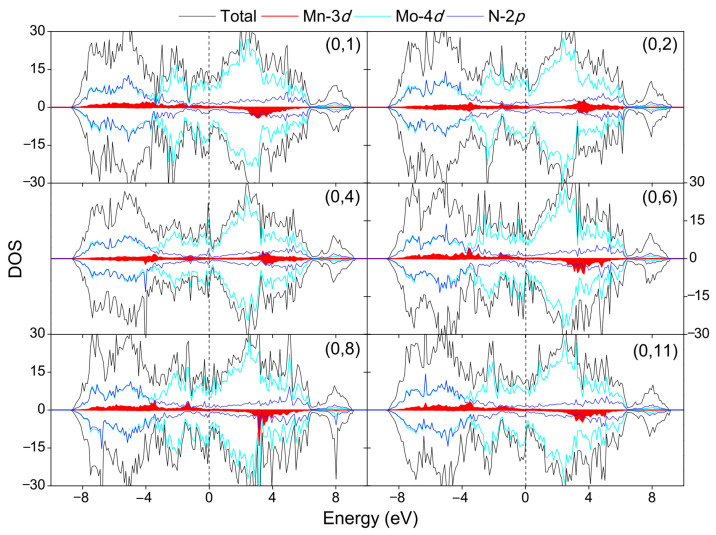
Total and partial DOS of the selected double-doped systems. The Fermi level is set as zero.

The interactions responsible for the magnetic ground state of the double Mn-doped systems can be theoretically estimated by calculating the interatomic-exchange constant *J*, by making use of the total energies in the ordered ferromagnetic (FM) and antiferromagnetic (AFM) structures, *E*_FM_ and *E*_AFM_. Based on the nearest-neighbor Heisenberg model, the value of *J* can be approximately deduced by Δ*E* = 4*JS*^2^, where Δ*E* = *E*_AFM_ − *E*_FM_ and *S* is the net spin per Mn dopant [46]. For a positive exchange parameter *J*, the ground state is FM, while the ground state is AFM at a negative exchange parameter *J*. Table 2 lists the main results of our work concerning the Mn doping for all the eleven (0, *n*) configurations, and the magnetic moment on each Mn atom is presented in the sixth column. It is seen that the magnetic moments of Mn atoms are almost independent of Mn distributions and the values are close to 4 *µ*_B_, implying that each Mn atom doping in the supercell possesses about +3e charge with a spin *S* = 2. Accordingly, the interatomic-exchange constants *J* were calculated and the variation of *J* value with the Mn–Mn distance is plotted in Figure 5. Interestingly, a complex oscillatory behavior of the *J* value as a function of Mn–Mn distance is observed. Among the eleven configurations, five positions ((0, 1), (0, 5), (0, 6), (0, 8), (0, 11)) result in FM and six positions ((0, 2), (0, 3), (0, 4), (0, 7), (0, 9), (0, 10)) result in AFM coupling between two dopant Mn atoms. The lowest energy configuration is found to be an FM state with a *J* value of 4.8 meV and Mn–Mn distance of 6.44 Å (configuration (0, 8)). Other configurations are higher than the ground state in energy by 68 to 422 meV (cf. Table 2). The oscillation of the *J* values in Figure 5 indicates that the different magnetic couplings of the Mn–Mn pair cannot be simply attributed to the different Mn–Mn distances. As for the case of FM orientation, the maximum *J* coupling is reached at the (0, 6) configuration with the Mn_0_–Mn_6_ separation of 5.77 Å. Although this Mn–Mn distance is longer than the nearest Mn_0_–Mn_1_ distance by 3.04 Å, the coupling strength of Mn_0_–Mn_6_ (*J* = 23.9 meV) is about 8.5 times stronger than that of Mn_0_–Mn_1_ (*J* = 2.8 meV). Compared with the FM orientation, the oscillation magnitude of *J* value for the AFM orientation is relatively weaker, and the largest *J* value (−3.3 meV) is achieved at the third-nearest Mn_0_–Mn_3_ configuration in which the Mn atoms are separated by 3.88 Å. The above evidence suggests that long-range magnetic coupling exists between the Mn–Mn pair in the supercell. The delocalized character of the Mn-3*d* states may offer some clues for this long-range magnetic interaction between the Mn dopants. As can be seen from the partial DOS of the double-doped systems in Figure 6, the Mn-3*d* states are relatively delocalized and strongly hybridize with the electronic states of the host atoms in the supercell, which translates into a long-range effective interaction between the dopant states.

Ferromagnetic materials are characterized by their coercivity (*H*_c_), the magnetic field necessary to reduce the magnetization to zero, and their Curie temperature (*T*_*C*_), the temperature at which random motions cause the magnetization to vanish [47]. Strong spin exchange coupling is a prerequisite for high coercivity [48]. This is because strong exchange interaction makes it hard to flip the spins under an applied magnetic field [48,49]. The strength of Mn−Mn spin exchange can be rationalized by the parameter *J*. A large positive *J* value (such as 23.9 meV of (0, 6) configuration) indicates a strong FM spin exchange coupling of the Mn–Mn pair, which may lead to a high coercivity. The Curie temperature *T*_C_ can be roughly estimated by means of the mean-field approximation and calculated according to the following equation [50]:

(2) $$T_{C}=\frac{2\Delta E}{3k_{B}}$$

where Δ*E* is the total energy difference between AFM and FM states and *k_B_* is the Boltzmann constant. Using Equation (2), the *T_C_* of (0, 1), (0, 6), (0, 8) and (0, 11) configurations are estimated to be higher than the room temperature; especially, the *T_C_* of (0, 6) configuration is up to 2951 K. Such high Curie temperatures make the material promising for practical applications.

**Figure 7:**
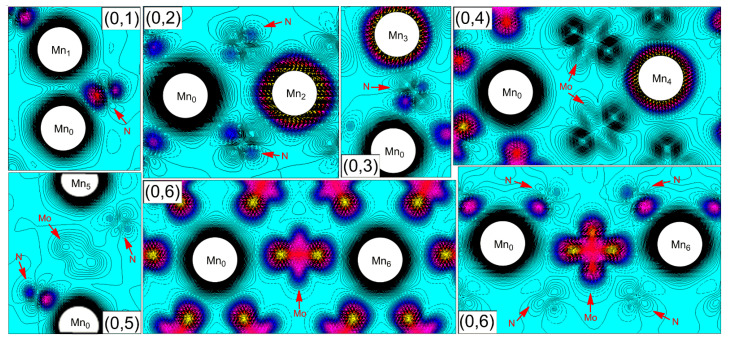
Spin charge density distribution on the planes containing the Mn–Mn pair and their mediating Mo and N atoms. Positive spin density is represented by solid lines, while negative spin density is represented by dashed lines and marked by colors.

To gain more insight into the magnetic nature of the double Mn-doped systems, we calculated the spin charge density distributions, as shown in Figure 7. For the (0, 1) configuration, the two Mn dopants are ferromagnetically coupled to each other with the nearest Mn–Mn distance of 2.73 Å considered in our calculations. We find that this Mn–Mn distance is comparable to that of the free Mn_2_ cluster (2.62 Å) [51]. Thus, a direct interaction of Mn–Mn *d*-orbital core spins is nonnegligible in the (0, 1) configuration, which gives rise to a bonding state between Mn_0_ and Mn_1_ (cf. Figure 7). Furthermore, the three N atoms, which simultaneously connect Mn_0_ and Mn_1_, contribute their 2*p* orbitals to form *p*–*d* hybridization with two Mn dopants. Evident overlap of N-2*p* and Mn-3*d* states could be observed from the partial DOS in Figure 6, demonstrating an indirect Mn–Mn exchange interaction mediated by the N atoms. This *p*–*d* exchange interaction between N and dopant Mn can also be confirmed by the magnetic moment of about −0.05 *µ*_B_ for the mediated N atoms. According to the Zener model, the direct interaction between *d* orbitals of adjacent Mn atoms tends to result in an AFM configuration of the *d* spins. Only when the indirect interaction dominates over the direct coupling between adjacent *d* orbitals is ferromagnetism present [52]. Therefore, the coupling of the Mn_0_–Mn_1_ pair is determined by a competition between the direct *d*–*d* interaction and the indirect *p*–*d* exchange. Due to the fact that Mn_0_ and Mn_1_ are ferromagnetically coupled, the indirect interaction of *d* states on different Mn atoms mediated by the *p* states of N atom is dominant in the (0, 1) configuration.

A different case is found for the configurations with larger Mn–Mn separations ((0, 2) to (0, 11)), in which the two Mn atoms are too far apart (more than 3.18 Å) to allow significant direct overlap of the orbitals and the direct *d*–*d* coupling between the Mn–Mn pair should be ignored. In such case, the key role underlying the mediated Mn–Mn interaction (FM or AFM) in these configurations should be sought in the electronic processes that take place in the segments connecting two Mn dopants. Here, the mediating segments for the Mn–Mn pairs in the (0, 2) to (0, 11) configurations can be divided into the following two cases:

Mn–N–Mn, (1)

and

Mn–N–Mo–N–Mn (2)

That is, in segment (1) the mediation takes place only by N atom, while in segment (2) the mediated segment is N–Mo–N.

We first focus on the case of the Mn–Mn pair mediated only by the N atom (i.e., two Mn atoms sharing a common N neighbor, forming the segment of Mn–N–Mn), which refers to the configurations of (0, 2) and (0, 3). An indirect Mn–Mn exchange interaction mediated by the N atom is dominant in the two configurations. As seen from Figure 7, the spin polarizations of the mediating N atoms in the two configurations are antiparallel to those of the Mn atoms, and *p* character of the spin-polarized N orbitals is evident. The mediating N atom provides two different *p*-orbitals to hybridize with Mn_0_ and Mn_2_ (or Mn_0_ and Mn_3_), respectively. That is, the spin-up N *p*-orbital makes a bond with a spin-down Mn orbital, and the spin-down N *p*-orbital bonds to a spin-up Mn orbital. Superexchange is naturally developed between the Mn dopants along the segment of Mn–N–Mn, thus resulting in the AFM alignment of Mn_0_ and Mn_2_ (or Mn_0_ and Mn_3_). Superexchange is a mechanism which describes an interaction between moments on atoms too far apart to be connected by direct exchange but coupled over a relatively long distance through the mediating atoms [53]. The extent to which a mediating atom orbital contributes to the overall interaction is governed by the magnitude of its orbital overlap with the coupled metal orbitals, which is influenced by the factors such as the symmetry and the distance between the mediating atom and metal. The weaker magnetic coupling of Mn_0_–Mn_2_ (*J* = –2.9 meV) as compared to that of Mn_0_–Mn_3_ (*J* = –3.3 meV) is mainly attributed to the nearly 90° exchange path (∠Mn_0_–N–Mn_2_ ≈ 95°) and the longer Mn–N distances in the mediating segment (the bond lengths of Mn_0_–N and N–Mn_2_ are about 0.12 Å and 0.07 Å longer than those of Mn_0_–N and N–Mn_3_, respectively).

The situation is more complicated in the case of the mediating segment (2), i.e., Mn–N–Mo–N–Mn, which corresponds to the configurations of (0, 4) to (0, 11). In this case, the mediated Mn–Mn interaction is mainly due to the electronic process that takes place in the segment of N–Mo–N connecting two Mn atoms. Detailed analysis reveals that the presence of Mo atom in the segment plays a critical role in mediating the Mn–Mn magnetic interaction. In fact, owing to the anisotropy of electronic structure and the directional nature of chemical bonding, the magnetic coupling of the Mn–Mn pair differs from one configuration to another. Here, we take (0, 4), (0, 5), and (0, 6) configurations as examples to illustrate the magnetic coupling mechanism of the Mn–Mn pair mediated by the segment of N–Mo–N.

We first pay our attention to the (0, 6) configuration, in which the two Mn atoms are strongly coupled with each other with a relatively large *J* value of 23.9 meV, indicating a strong FM interaction of Mn_0_–Mn_6_ pair. From Figure 4, we notice that the two Mn dopants in the (0, 6) configuration are both located at the *ab* plane of the supercell, and each substitutional Mn atom is surrounded by six Mo atoms, which forms a slightly deformed hexagon structure. It is evident from Figure 7 that the six surrounding Mo neighbors are all antiferromagnetically coupled to the Mn dopant, and notable magnetic moments locate at the Mo sites, giving rise to an AFM order in the neighborhood of the Mn atom. This AFM environment around the Mn dopant is the origin of the reduction of total magnetic moment, as reflected from Table 2. In particular, the induced magnetic moment of the mediating Mo atom (connecting Mn_0_ and Mn_6_) is up to −0.54 *µ*_B_. The large magnetization of the mediating Mo atom is partially due to the indirect superexchange interaction mediated by the N atom that connects Mn and Mo, i.e., two different N *p*-orbitals antiferromagnetically hybridize with Mn and Mo, respectively, leading to the induced spin of Mo that is antiparallel to that of Mn. This indirect Mn–N–Mo superexchange interaction resembles the case of Mn–N–Mn, as discussed above. Most importantly, the significant spin polarization of the Mo atom is arising from the direct interaction between the Mo-4*d* and Mn-3*d* orbitals, which can be clearly seen from the spin charge density distributions in Figure 7. The three transition metal *d*-orbitals (one Mo-4*d* orbital and two Mn-3*d* orbitals) along the chain of Mn–Mo–Mn couple well with each other to reduce the total energy. The large *J* value of the (0, 6) configuration is good proof that the direct *d*–*d* interaction between the dopant Mn and the mediating Mo atom is beneficial to enhance the superexchange interaction of the Mn–Mn pair.

Although the Mn_0_–Mn_4_ pair in the (0, 4) configuration both locate at the *ab* plane as well, the local symmetry around the doping site and the spin polarization of the neighboring Mo atoms to the Mn dopants are different from those of the (0, 6) configuration. Because of the loss of symmetry at the doping site upon geometry optimization, the two mediating Mo atoms move away from their original positions and connect Mn_0_ and Mn_4_ with nonequivalent Mn–Mo distances (i.e., the Mn_0_–Mo distance is more than 3.18 Å, while the Mn_4_–Mo distance is less than 2.72 Å). It is seen from Figure 7 that the magnetic moments of two mediating Mo atoms are antiparallel to the nearer Mn dopant (Mn_4_), and, at the same time, are parallel to the farther Mn dopant (Mn_0_). For the two mediating Mo atoms, the direct *d–d* interaction to Mn_4_ is significant, but it is really weak to Mn_0_. The interaction between Mn_0_ and the mediating Mo atom is mainly mediated by the N atoms. Such nonequilibrium interactions along the mediating segment consequently lead to the AFM coupling of the Mn_0_–Mn_4_ pair. By examining the spin charge density distributions of (0, 4) and (0, 6) configurations, we find that the exhibited magnetism of the Mo atoms parallel/antiparallel to that of the Mn dopants is not only correlated with the longer/shorter Mn–Mo distances, but also the coordination orientation of Mo *d*-orbital that participates in bonding.

As for the (0, 5) configuration, the two Mn dopants are FM coupled to each other along the *c* direction of the supercell. It is visible from Figure 7 that this long-ranged FM coupling of the Mn_0_–Mn_5_ pair is established by the indirect *p*–*d* exchange between the N *p*-orbital and the transition metal *d*-orbitals along the chain of Mn(↑)–N(↓)–Mo(↑)–N(↓)–Mn(↑). One Mn dopant (Mn_0_) develops a strong bonding with its nearest N atom and induces AFM polarization of the N atom with a magnetic moment of about −0.05 *µ*_B_, which further leads to a 0.13 *µ*_B_ spin of the mediating Mo atom, reflecting the effect of through-bond spin polarization. The other Mn dopant (Mn_5_) in turn couples to the mediating atoms (N and Mo) in the same way for an energy gain, resulting in an indirect FM coupling among the two Mn dopants. However, due to the weak hybridization between Mo *d*-orbital and N *p*-orbital along the *c* axis, the mediated interaction through the segment of N–Mo–N in (0, 5) configuration is rather feeble, which leads to a relatively small *J* value of 0.7 meV.

As stated in the above, the mediated Mn–Mn interaction should be sought in the electronic processes that take place in the segment that connects two Mn dopants. From the observations of (0, 4), (0, 5), and (0, 6) configurations, we find that the spin of the mediating N atom (which makes a bond to the Mn dopant) is always antiparallel to that of Mn regardless of the Mn doping position. The mediating Mo atom can couple to the Mn dopant either ferromagnetically or antiferromagnetically, which mainly depends on the different coupling mechanisms (*p–d* exchange, superexchange, and *d–d* coupling). The *p–d* exchange mediated by the N atom could induce the Mo–Mn FM coupling, while the superexchange through the N atom can result in the Mo–Mn AFM coupling. The substantial *d–d* coupling between Mo and Mn could effectively enhance the magnetic coupling of the Mn–Mn pair, which plays a critical role in mediating the Mn–Mn indirect interaction. The above analysis provides some insight into the origin of magnetic coupling between the Mn dopants. For the configurations in which the two Mn dopants are connected by the segment of N–Mo–N (configurations of (0, 4) to (0, 11)), the indirect FM/AFM Mn–Mn interaction can be described as follows: One Mn dopant induces AFM polarization of its neighboring N atom (one of the N atoms belongs to Mn–N–Mo–N–Mn), and this spin-polarized N atom may polarize the mediating Mo atom in a parallel way or an antiparallel way (through *p–d* exchange or superexchange). Then, by the similar mechanism, the mediating Mo atom interacts with the other N atom connected to it, and, through which, ferromagnetically or antiferromagnetically couples to the other Mn dopant. That is, one of the Mn dopants dictates the spin polarization of its neighboring N atom, then this N atom affects the spin direction of the mediating Mo atom, and, through which, interacts with the other N atom, and eventually determines the FM or AFM coupling of the Mn–Mn pair. The different coupling mechanisms, i.e., the *p–d* exchange and the superexchange mediated by the N atom, and the *d–d* coupling between Mo and Mn, are associated with the geometry structure along the segment (such as bond length and bond angle) and the coordination orientation of the bonding orbital. Additionally, it should be emphasized that, except for the mediating atoms, other host atoms located next to the dopants also play important roles: first they become spin-polarized and then they can polarize their nearest neighbors, which facilitates the development of delocalized spin-polarized orbitals over a more extended range.

## 4. Conclusions

Based on the density functional theory, the structural, electronic, and magnetic properties of *δ*-MoN with one Mn atom substituted at different Mo sites (2*a* and 6*c* in the International Tables) were firstly studied. The substitution of Mn atom results in the local distortion of the structure and the shrink of the unit cell. Formation energy calculations confirm that it is energetically favorable to form Mn substitution in *δ*-MoN. It is noteworthy that the Mn dopants located at 2*a* and 6*c* sites lead to significant spin splitting of the density of states and induce 2.74 and 2.56 *μ*_B_ total magnetic moments, respectively, suggesting that the Mn dopant induces magnetism in *δ*-MoN. The calculations were then extended to a 2 × 1 × 2 supercell, which contains two impurity Mn atoms, to investigate the favorable coupling between the Mn dopants. Among the eleven different magnetic configurations, five positions result in FM and six positions result in AFM coupling between the Mn–Mn pair. Strong evidence indicate that long-range magnetic coupling exists between the Mn dopants in the supercell. The different couplings of the Mn–Mn pair cannot be simply attributed to the different Mn–Mn distances, but closely related to the electronic processes that take place in the mediating segment (–N– or –N–Mo–N–) that connects two Mn dopants. The mechanisms responsible for the FM/AFM coupling of the Mn–Mn pairs are the superexchange and the *p*–*d* exchange mediated by the N atoms, and the *d*–*d* coupling between the Mo and Mn atoms.

## Figures and Tables

**Table 1:** Optimized lattice parameters of *δ*-MoN before and after Mn substitution, and formation energies of Mn-MoN(2*a*) and Mn-MoN(6*c*).

Configurations	*a* (Å)	*c* (Å)	*c*/*a*	Volume (Å^3^)	*E*_f_ (eV)
*δ*-MoN	5.757	5.668	0.985	162.7	
Mn-MoN(2*a*)	5.726	5.626	0.983	159.5	−1.71
Mn-MoN(6*c*)	5.714	5.644	0.988	159.6	−1.68

**Table 2:** The relaxed Mn–Mn distance (*d*), the energy difference (Δ*E*) between FM and AFM states, the energy relative to the ground state (Δ*E*_ground_), the total magnetic moment of the supercell (*M*_total_), the magnetic moments of two Mn dopants (*M*_1_ and *M*_2_), and the formation energies (*E*_f_) for various double Mn-doped configurations.

Configurations	*d* (Å)	Δ*E* (meV)	Δ*E*_ground_ (meV)	*M*_total_ (*μ*_B_)	*M*_1_/*M*_2_ (*μ*_B_)	Coupling	*E*_f_ (eV)
(0, 1)	2.73	44	283	4.49	3.88/4.01	FM	−3.20
(0, 2)	3.18	−46	422	−0.34	4.03/−4.05	AFM	−3.06
(0, 3)	3.88	−52	265	−0.41	4.01/−4.05	AFM	−3.22
(0, 4)	5.01	−39	236	0.07	4.08/−4.06	AFM	−3.25
(0, 5)	5.65	11	210	3.93	3.98/3.98	FM	−3.27
(0, 6)	5.77	382	68	3.67	3.99/3.99	FM	−3.41
(0, 7)	6.07	−19	381	0.05	4.05/−4.06	AFM	−3.10
(0, 8)	6.44	77	0	3.38	4.00/4.00	FM	−3.48
(0, 9)	6.47	−15	376	−0.35	4.03/−4.06	AFM	−3.11
(0, 10)	7.25	−36	220	−0.96	4.00/−4.03	AFM	−3.26
(0, 11)	8.08	54	146	3.50	3.99/3.99	FM	−3.34

## Data Availability

Not applicable.
